# Comparison between two divergent diets, Mediterranean and Western, on gut microbiota and cognitive function in young sprague dawley rats

**DOI:** 10.1080/29933935.2024.2439490

**Published:** 2024-12-18

**Authors:** Rebecca J. Solch-Ottaiano, Elizabeth B. Engler-Chiurazzi, Colin Harper, Savannah Wasson, Sharon Ogbonna, Blake Ouvrier, Hanyun Wang, Madison Prats, Katherine McDonald, Ifechukwude J. Biose, Lori A. Rowe, MaryJane Jones, Chad Steele, Gregory Bix, Demetrius M. Maraganore

**Affiliations:** a Clinical Neuroscience Research Center, Department of Neurology, Tulane University School of Medicine, New Orleans, LA, USA; b Tulane Brain Institute, Tulane University, New Orleans, LA, USA; c Clinical Neuroscience Research Center, Department of Neurosurgery, Tulane University School of Medicine, New Orleans, LA, USA; d Virus Characterization, Isolation, Production and Sequencing Core, Department of Microbiology, Tulane National Primate Center, Covington, LA, USA; e Department of Microbiology and Immunology, Tulane University School of Medicine, New Orleans, LA, USA

**Keywords:** Mediterranean diet, Western diet, gut microbiota, memory, cognitive function

## Abstract

Clinical studies strongly suggest the importance of diet quality on cognition in youth populations (15–24 years). The Mediterranean diet (MeDi) has been shown to improve cognition in contrast to the commonly consumed Western diet (WD). The gut microbiota may serve as a mechanism for diet-induced changes in cognition. Ten-week-old male Sprague Dawley rats were assigned a MeDi or WD (*n* = 10/group) for 14 weeks. Prior to neurobehavior assessments, microbiota community composition was assessed. At the genus level, the relative abundance of four bacteria increased with the MeDi and five decreased compared to the WD. Rats in the MeDi group demonstrated cognitive flexibility and improvement in reference and working memory relative to the WD group. At the end of the study, serum cytokines were increased, and low-density lipoproteins were decreased in the MeDi group. Markers for neuroinflammation, blood-brain barrier, glial cells, and synaptic plasticity in brain regions did not differ between groups. Overall, the MeDi modulated gut microbiota, cognitive function, and serum lipid and cytokines but not gene expression in the brain compared to the WD. Further studies are needed to determine causality between diet-modulated gut microbiota, cognitive function, and immune function.

## Introduction

Dietary patterns are intertwined with health outcomes across the human lifespan. Diet is particularly important for brain development in adolescents (10–19 years) and youth (15–24 years). Unfortunately, most adolescents are adhering to a Western diet (WD) pattern exhibited by consuming less than five food groups per day, with the highest prevalence of consumption being grains, milk products, and meats.^1^ This contributes to the adherence to a poor quality diet ranging from 39.8–66.6% in youth aged 2 to 19 years, with an increase in poor diet quality adherence as age increases.^2^ Adherence to the WD in youth has been associated with poor cognitive function.^3–6^ By contrast, adherence to nutrient-dense diets such as the Mediterranean diet (MeDi), low in saturated fats and high in fiber from an increase in plant foods and fresh fruit,^7^ is associated with improved cognitive performance in youth with good and average adherence compared to poor adherence.^8^

The mechanisms by which diet affects cognitive function are multifold and not fully understood. One potential mechanism is through modulation of the gut microbiota, as diet directly shapes gut microbiota composition. Accumulating evidence supports the involvement of the gut-brain axis in cognitive function.^9–11^ Several factors, including environment (i.e., diet, stress, physical activity) and genetics, impact the gut microbiota.^12^ However, the largest modulator of gut microbiota is diet, accounting for up to 57% of the variability.^13,14^ Studies have demonstrated that a diet high in fat results in a perturbed microbiota composition that precedes cognitive decline.^15,16^ Diet-modulated gut microbiota may impact cognition through immune dysregulation, as unhealthy diets such as the WD result in inflammation in both the gut and brain regions important for memory and learning.^17,18^

Strategies aimed at discerning the relationship between diet and cognitive function and related mechanisms have relied on the use of high-fat and chow diets. This has provided a fundamental understanding of the detrimental impact of a diet high in fat compared to a laboratory diet, however, consideration should be taken for the intricacies of diet, including the source of macronutrients such as lipids. Findings from these foundational studies on high-fat and chow diets, are not fully translatable to the WD dietary patterns typically followed by humans^19^ or a MeDi, respectively. Recently, pre-clinical studies have begun to explore the impact of interventional diets such as MeDi compared to the WD on health outcomes.^20–24^

To build upon this, and in order to mimic human and non-human primate study designs where the MeDi is the intervention and is compared to the typically consumed WD (control),^21,23,25–27^ we chose not to include chow in our experimental design. Therefore, we explored a 14-week MeDi intervention on fecal microbiota composition, cognitive function, and physiological outcomes relative to a WD, for the first time in young adult male rats.

## Methods

### Animals and diets

One independent study was conducted with eight-week-old male Sprague-Dawley rats purchased from Envigo (Saint Louis, MO, USA) and acclimated for two weeks before the start of the study. The rats were placed in sterile housing in a climate-controlled room on a 12-hour light/dark cycle with ad libitum food and water. Rats (*n* = 10/group) were randomly assigned using a random number generator available in Excel (Microsoft) to either the WD (15% protein, 50% carbohydrate, 35% fat; Research Diets #D12052705C) or the MeDi (12% protein, 45% carbohydrate, 43% fat; Research Diets #D12052702D). The WD was based on the US Department of Agriculture’s 2008 Dietary Assessment of Major Food Trends and the traditional MeDi was based on the Food and Agriculture Organization’s Food Balance Sheets from Greece in 1961.^20^ While percentages of macronutrients are similar between diets, the primary ingredient sources differ. For example, the primary fat source for MeDi is olive oil, and the WD is butter. Dietary sources for each diet can be found in Table 1. The omega-6:omega-3 ratio for the MeDi was 2:1 and the WD was 11:1. The MeDi contained 63.4 g/kg of fiber (cellulose, 47.6; inulin 15.8) while the WD contained 27.9 g/kg of fiber (cellulose, 21.0; inulin, 6.9). Diets were kcal (4.3 kcal/g), vitamin, and mineral matched.

**Table 1.** Composition of the Mediterranean and Western diets as g/kg.^a^

	MeDi^b^	WD^c^
Casein	51.43	44.50
Fish Protein Isolate	31.56	9.80
Egg White	10.52	63.60
Beef, Cooked, Powdered, 5013	72.35	88.90
L-Cystine	3.51	3.50
Corn Starch	0.00	34.70
Wheat Starch	426.65	225.50
Potato Starch	0.00	34.70
Sucrose	71.30	237.10
Fructose	22.21	25.40
Cellulose, BW200	47.57	21.0
Inulin	15.78	6.90
Corn Oil	0.00	39.80
Menhaden Oil (200 ppm tBHQ)	15.43	1.20
Palm Kernel Oil	11.57	0.00
Butter, Anhydrous	8.65	62.60
Flaxseed Oil	7.13	1.20
Olive Oil	137.58	31.80
Cellulose	47.60	21.0
Inulin	15.80	6.90
Mineral Mix S10026	11.69	11.60
Dicalcium Phosphate	15.20	15.00
Calcium Carbonate	6.43	6.40
Potassium Citrate, 1 h2O	19.29	19.10
Vitamin Mix V10001	11.69	11.60
Biotin (1%)	0.02	0.10
Choline Bitartrate	2.34	2.30
Cholesterol	0.00	1.70
Resveratrol (50% Trans Resveratrol)	0.05	0.00
FD&C Red Dye #40	0.06	0.00
FD&C Blue Dye #1	0.00	0.11

^a^Diets were kcal (4.3 kcal/g), vitamin and mineral matched.

^b^The MeDi (Research Diets #D12052705C) was 12% protein, 45% carbohydrate, and 43% fat.

^c^The WD (Research Diets #D12052702D) was 15% protein, 50% carbohydrate, and 35% fat.

MeDi, Mediterranean diet; WD, Western diet.

Diets were kcal matched to control the independent effects of obesity, as obesity is a risk factor for cognitive impairment.^28–30^ Body weights were measured weekly, and food consumption, as an indicator for energy intake, was measured biweekly throughout the study. Power was lost in the vivarium on two separate occasions due to uncontrollable circumstances, city infrastructure failure, and a natural disaster (hurricane) for two and three days, respectively. To allow animals to return to normal conditions, study tasks were delayed for 16 days ± 2 days. In totality, this was a 20-week study with animals consuming diets for 14 weeks before beginning behavioral assessments from weeks 14–17, followed by euthanasia at week 20. Animals were monitored by veterinary staff during these outages and dietary interventions continued. All procedures were approved by Tulane University Institutional Animal Care and Use Committee and adhered to NIH standards.

### Microbial analysis

#### DNA extraction and 16S sequencing

Fresh fecal samples were collected at baseline (Week 0) and before behavioral assessments (Week 14) using aseptic technique and stored in a −80°C freezer until analysis. Fecal DNA was extracted using Quick-DNA™ Fecal/Soil Microbe Microprep Kit (Zymo Research, Irvine, CA) according to the manufacturer’s protocol. After DNA extraction, the Illumina 16S metagenomic sequencing library preparation guide was followed and the 16S V3 and V4 gene region were amplified using forward (5ʹ-TCG TCG GCA GCG TCA GAT GTG TAT AAG AGA CAG CCT ACG GGN GGC WGC AG-3ʹ) and reverse (5ʹ-GTC TCG TGG GCT CGG AGA TGT GTA TAA GAG ACA GGA CTA CHV GGG TAT CTA ATC C-3ʹ) primers. The samples were then multiplexed with the Illumina Nextera UDIs. The resulting libraries were sequenced on an Illumina MiSeq using the MiSeq v3 (600 cycle) reagent kit. Raw pair-ended 16s rRNA sequences were demultiplexed and filtered based on their quality by Quantitative Insights Into Microbial Ecology (QIIME) 2 framework core function.^31^ Reads were de-noised and merged by DADA2 pipeline.^32^ Sequences were clustered at the 99% identity level, and taxonomies were determined using SILVA 138.^33,34^ An operational taxonomic unit table and phylogenetic tree were constructed using QIIME2 to be used for statistical analysis.^31^ Microbial data were analyzed using R program (2021.09.1). Alpha diversity indices including Chao1 (richness), Simpson (evenness), and Simpson (richness and evenness) were calculated using the R package Vegan.^35^ Kruskal-Wallis rank-sum test was used to compare alpha diversity for each index between diets. Differences in principal coordinate analyses (PCoA) were analyzed via permutational multivariate analysis of variance with the Adonis function in the vegan package using Bray-Curtis, weighted UniFrac, and unweighted UniFrac distances. Differences in relative abundance between diets at the phylum, class, order, family, genus, and species level were determined by the linear discriminant analysis (LDA) effect size (LEfSe) method with a false discovery rate (FDR) of 0.1 using the R package MicrobiotaProcess.^36^

### Behavioral assessments

A battery of neurobehavioral assessments was used to test aspects of short- and long-term learning and memory. Tests were given in ascending order of cognitive demand. All behavioral testing took place between 0700 and 1400 hrs in a dimly lit room. After consuming the diets for 14 weeks, behavioral tests were completed from weeks 14–17.

For all tests, spatial cues consisted of six different shapes placed around the apparatuses. A video camera above the mazes was used to track the animals using a software program (AnyMaze, 6.35) for all tests.

#### Y-maze

The spontaneous alternation Y-maze was utilized to capture short-term recognition memory.^37^ Animals were acclimated to the room for 60 min before testing. The Y-maze apparatus (Stoelting, Wood Dale, IL) consists of three closed arms (10 cm wide, 50 cm long, 20 cm high) connected in the shape of a Y. One at a time, animals were placed in the start arm and allowed to explore freely for 10 min. Entry into each arm was recorded. A correct alteration score was calculated as the number of times the three arms were sequentially entered. Total alterations were derived as the number of total arm entries minus two. The percent correct alterations is the number of correct alterations divided by the total number of alterations made.

#### Morris water maze

To test spatial reference memory, the Morris water maze (MWM) was utilized. A tank (1.8 m diameter) filled with room temperature water and colored black with nontoxic paint for all water maze testing. The apparatus was hypothetically divided into four quadrants (North, East, South, and West). A hidden platform was placed in the Northeast quadrant and remained in this fixed location throughout the duration of the testing. Each day the animal was placed in the maze from any of the four start locations and had 60 s to locate the hidden platform. Animals were tracked using the ANY-maze video tracking software and cameras to ensure unbiased data collection. If an animal did not locate a platform, it was gently led to it by the investigator. Once found, animals stayed on the platform for 15 s and were returned to a heated cage until the subsequent trial. The animals were given four trials per day for five days. On the fifth day, animals were given an additional 60 s probe trial where the platform was removed. This trial evaluated whether the animals localized the platform to the spatial location, as animals who had learned the platform location were expected to spend the greatest percent distance in the target quadrant.^38,39^ Probe trial data were analyzed for group differences in percent time spent in the target quadrant minus percent time spent in the opposite quadrant. To assess cognitive flexibility in learning and perseverance, animals underwent two days of reversal testing following day five. The platform was placed in the opposite quadrant (southwest). Animals underwent four trials per day for two days and, on the last day, had a 60 s reversal probe trial.

#### Water radial arm maze

An eight-arm water radial arm maze (WRAM) was used to measure discrete components of memory, including spatial working and reference memory. A tank (1.8 m diameter) filled with room temperature water and colored black with nontoxic paint was used for all water maze testing. Escape platforms were placed at the end of four arms approximately 1 cm below the water. Animals were randomly assigned to one of the four arm combinations containing platforms, and this assignment remained constant throughout the experiment. All animals were released from the same start arm with their body facing the tank wall and had 90 s to reach an escape platform. If an animal did not find a platform within the 90 s, it was led to the nearest platform. Once on the platform, animals remained on it for 15 s and then were returned to a heated cage for 30 s. Between an animal’s trial, the just-chosen platform was removed. A daily session consisted of these events until all four platforms were located. Therefore, a daily session consisted of four trials per session for each animal. Each animal was given one session per day for ten consecutive days. Day 1 was considered training because the animal had no previous experience in the maze. Days 2–10 were testing sessions. Arm entries were considered when the animal’s entire body entered the arm and was collected by a single investigator. Independently, two different investigators quantified discrete error types for each daily session as done previously.^38^ Working memory correct (WMC) errors were the first and repeated entries into any arm from which a platform had been removed during that day’s session. Reference memory errors were the first entries into any arm that never contained a platform. Working memory incorrect (WMI) errors were repeat entries into reference memory arms. Trial one WMC errors were not included in the analysis as it is not possible to make a WMC error on the first trial of each day. Total errors were calculated as the sum of the three error types. On days 11–12, a 24-hour delay was imposed between trials 2 and 3 to assess the retention of multiple items of spatial information. The dependent measure for performance on the delay day was total errors on trials 3 and 4, the trials after the 24-hour delay.^38^

#### Visible platform

As the MWM and WRAM tests rely on proper eyesight to appropriately learn the spatial tasks, a visible platform test was performed at the end of the study using the tracking system. Briefly, spatial cues around the room were removed, and a visible platform (painted white to contrast the black water with a small flag attached to the platform) was placed approximately 2 cm above the water in a fixed location. Animals underwent six trials where they were placed in the maze from four different start locations. Swim distance and time to reach the platform was evaluated to determine any sight-dependent learning disability.

### Cholesterol analysis

After neurobehavioral assessments were completed (week 17), a natural disaster-induced power outage occurred resulting in a delay in tissue collection. As such animals were euthanized at week 20 (Methods 1.1 Animals and Diets). Blood was collected via cardiac puncture, allowed to coagulate at room temperature for 30 mins, placed on ice for at least 30 mins to allow further clotting, and then centrifuged at 1,000 × g for 10 mins at 4**°**C. Cholesterol levels, high-density lipoprotein (HDL), and low-density lipoprotein (LDL) were determined using a cholesterol quantification kit (MAK043, Sigma-Aldrich) according to the instructions of the manufacturer.

### Cytokine multiplex

For cytokine analysis, thawed serum samples (*n* = 7–10/diet) were clarified by centrifugation at 1,000 × g for 10 mins at 4**°**C prior to the assay. Serum samples were assessed for protein levels of 23 cytokines using a Bio-Plex Pro™ Rat Cytokine Assay (Bio-Rad Cat# 12005641) according to the manufacturer’s instructions. The assay was read on a Bio-Plex® 200 Reader (Luminex 200) using the high PMT, R1 setting to collect the median fluorescence intensity. Using the Bio-Plex Manager® Software (Bio-Rad Laboratories, Hercules, CA), data were analyzed using a 5-parameter logistic curve and concentrations (pg/ml) were calculated.

### qRT-PCR

The hippocampus and striatum were homogenized in Trizol Lysis Reagent. RNA was extracted according to the RNA extraction kit manufacturer instructions (RNeasy Plus Mini Kit; Qiagen). RNA was converted to cDNA using iscript reverse transcriptase master mix (Bio-Rad). Gene expression was conducted with QuantStudio 3-Real-Time PCR Systems (Life Technologies) using TaqMan PCR Master Mix and specified probes (Thermo Fischer Scientific) for Claudin-5 (Rn01753146_s1), Occludin (Rn00580064_m1), intercellular adhesion molecule 1 (ICAM; Rn00563627_m1), vascular cell adhesion molecule 1 (VCAM; Rn00564227_m1), ionized calcium binding adaptor molecule 1 (IBA-1; Rn03993468_g1), triggering receptor expressed on myeloid cells 2 (TREM2; Rn01512170_m1), glial fibrillary acidic protein (GFAP; Rn01253033_m1), vimentin (Rn00667825_m1), IL-1α (Rn00566700_m1), IL-1β (Rn00580432_m1), IL-6 (Rn01410330_m1), C-X-C motif chemokine 10 (CXCL10; Rn01413889_g1), C-X-C motif chemokine receptor 3 (CXCR3; Rn02134090_s1), postsynaptic density protein-95 (PSD95; Rn00571479_m1), brain-derived neurotrophic factor (BDNF; Rn02531967_s1), and hypoxanthine phosphoribosyltransferase 1 (HPRT1; Rn01527840_m1; housekeeping gene). Data were analyzed comparing the WD group (control) to the MeDi group and presented as fold change of WD.

### Statistical analysis

Where appropriate, data were analyzed by student’s t-test for test-specific outcomes between dietary groups. Two-way repeated-measures ANOVA with Diet and Day or Trial in the model were performed for behavioral assessments with multiple trials. Šídák post-hoc adjustments for pairwise comparisons were applied where appropriate. All body weight, kcal consumption, and behavioral results were analyzed using Prism software (GraphPad Software, Inc., www.graphpad.com.) with statistical significance predefined as *p* < 0.05. Pearson correlation was used to determine the association between the relative abundance of taxa and behavioral or cytokine outcomes significantly different between diets. An FDR of 0.1 was applied to the Pearson correlation *p* values. Results are represented as mean ± SEM.

## Results

### Energy intake, weight, and lipid profile

**Figure 1.**
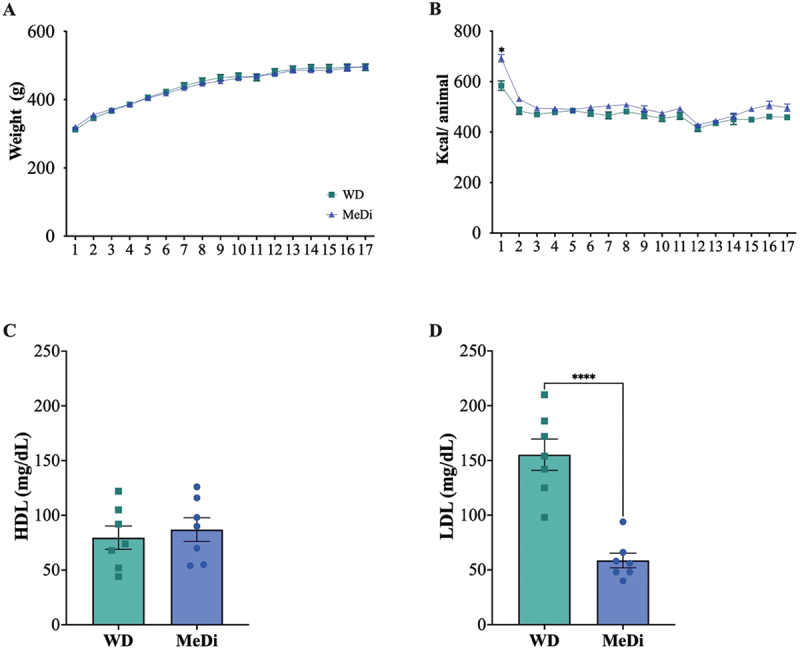
Weight in grams (A) did not differ between diet groups. Kilocalories (kcal) consumed did not differ between diets after week 1 (B). Serum high-density lipoprotein (C; HDL) did not differ between diets. However, serum low-density lipoprotein (D; LDL) concentration was higher in the WD group compared to the MeDi group. Kcal consumption was calculated per animal: [(grams consumed * kcal/g of diet)/2)]. Two-way repeated measure ANOVA with diet, week, and their interactions were performed with the šídák method for multiple comparisons for post hoc testing for body weight and kcal. Student’s t-test was performed for HDL and LDL values. Data are represented as mean ± S.E.M. *p < 0.05 MeDi vs WD; ****p < 0.0001. MeDi, Mediterranean diet (*n* = 7–10); WD, Western diet (*n* = 7–9).

Weight and Energy intake (kcal) were monitored throughout the experiment. Animals gained weight across the study (*p* < 0.001) but did not differ between the MeDi and WD groups (Figure 1A). There was no difference in energy intake (Figure 1B) between diets. This was anticipated as diets were kcal-matched to account for the independent effects of obesity on cognitive function.^28–30^ At Week 1, the MeDi group consumed more kcals compared to the WD group (*p* = 0.002) but did not differ for the remaining study weeks (Figure 1B). Assignment of animals to the MeDi had no effect on HDL serum levels (Figure 1C). However, compared to the WD, the MeDi group had significantly lower LDL serum levels (*p* < 0.0001; Figure 1D).

### Gut microbiota composition

Fecal samples were collected at baseline (Week 0) and prior to behavior assessments (Week 14) to reveal diet effects on gut microbiota composition. Samples were collected prior to behavior assessments to allow for potential correlations between gut microbial changes and cognitive function outcomes. When visually inspecting graphs of phylum-level relative abundance, one sample was identified as an outlier. This outlier was confirmed using the ROUT method on the relative abundance of Actinobacteria (Supplementary Figure S1). Additionally, the value was two standard deviations above the mean (mean = 0.18, standard deviation = 0.16, value = 0.62). This animal was excluded from all analyses.

**Figure 2.**
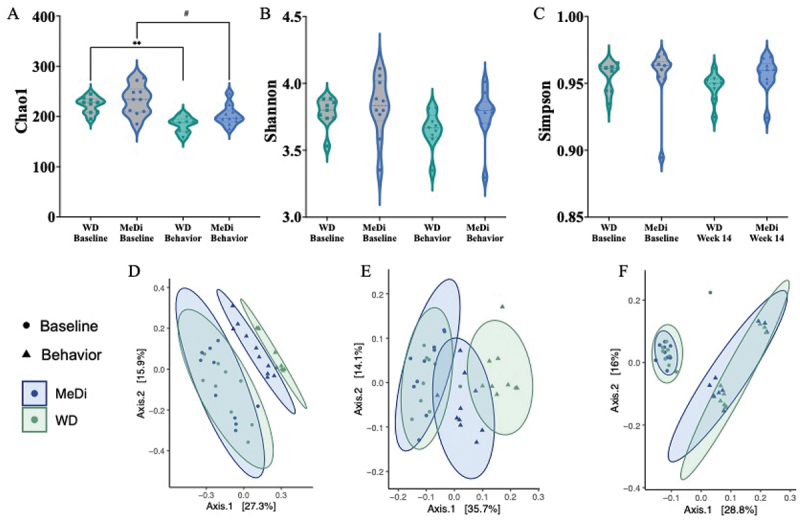
At baseline or before behavior, there were no differences in measures of ⍺-diversity including Chao1 (A), Shannon (B), or Simpson between the MeDi and WD groups (C). Diet groups did not differ at baseline, but before behavioral analyses (14 weeks of diet consumption) microbial composition was distinct between diets determined by Bray-Curtis dissimilarity (D) and weighted UniFrac (E), but not for unweighted UniFrac (F). Kruskal-Wallis Rank Sum Test was used for differences in ⍺-diversity and data are represented as interquartile range. Permutational multivariate analysis of variance using distance matrices was used to determine differences in β-diversity. Each data point represents an individual animal, the color represents the diet, and the symbol represents the timepoint. Ellipses represent a 95% confidence level. MeDi, Mediterranean diet (*n* = 7–10); WD, Western diet (*n* = 7–9).

For measures of ⍺-diversity, we assessed Chao1, Simpson, and Shannon indices for measures of richness (number of taxa), evenness (the relative abundances of those taxa), and richness and evenness, respectively (Figure 2A-C). Groups did not differ for any measurement at baseline when consuming a chow diet. After consuming the diets for 14 weeks, WD and MeDi groups had a trend to or decreased in Chao1 index (MeDi, *p* = 0.06; WD, *p* = 0.004), but not for Shannon or Simpson index compared to baseline. As Chao1 gives more weight to low-abundance taxa compared to Shannon and Simpson indices, this demonstrates that the MeDi and WD groups had fewer rare taxa compared to baseline but were not different between diets.^40^ Prior to behavior assessments (Week 14), there was no difference between the WD and MeDi groups for ⍺-diversity.

β-diversity was used to assess the variability in community composition (Diet). β-Diversity measurements included Bray Curtis dissimilarity, unweighted UniFrac distance (presence vs absence of taxa), and weighted UniFrac distance (accounting for the relative abundance of taxa) to determine the similarity or dissimilarity among samples. There were no differences at baseline between groups when consuming the chow diet (Figure 2D-F). There was a significant effect of Time (Baseline vs Behavior, *p* = 0.001) for all β-diversity measurements, indicating modulation of the gut microbiota by the WD and MeDi compared to a chow diet.

Prior to behavior assessments, there was a significant difference between the MeDi and WD groups for Bray Curtis dissimilarity (*p* = 0.001) and weighted UniFrac- (*p* = 0.001) but not unweighted UniFrac. Therefore, the diets may promote phylogenetically different microbes but not low-abundant taxa as Bray Curtis Dissimilarity and weighted UniFrac is significant, but not unweighted UniFrac. This may be in part due to similar food sources but varying amounts in the diets.

**Figure 3.**
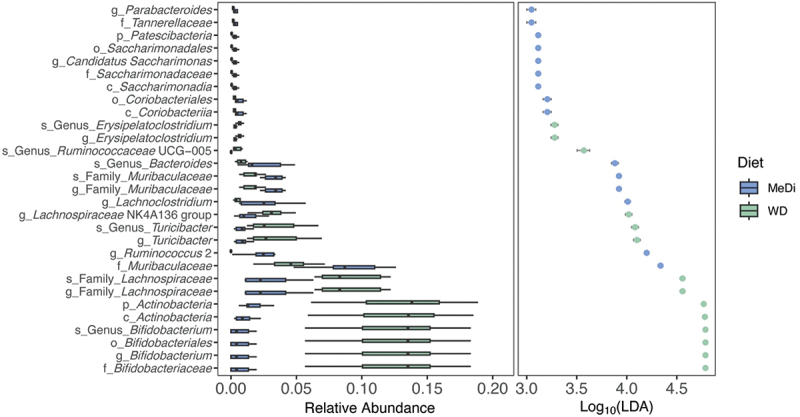
There were gut microbial changes across taxonomic levels between the MeDi and WD groups. Relative abundance and linear discriminant analysis (LDA) values were determined via LEfSe. Relative abundance is represented as an interquartile range. MeDi, Mediterranean diet (*n* = 10); WD, Western diet (*n* = 9).

Consumption of the MeDi induced widespread changes in the gut microbial community at various taxonomic levels compared to the WD (Figure 3 and Supplementary Table S1). At the phylum level, there was an increase in the relative abundance of *Patescibacteria* (*p* = 0.003, FDR = 0.08) and a decrease in *Actinobacteria* (*p* = 0.0002, FDR = 0.03) in the MeDi group. At the genus level, four genera increased in the MeDi group, including *Candidatus Saccharimonas* (*p* = 0.003, FDR = 0.08), *Lachnoclostridium* (*p* = 0.003, FDR = 0.08), *Ruminococcaceae* (*p* = 0.0001, FDR = 0.04), and *Muribaculaceae* (*p* = 0.001, FDR = 0.04). Four genera decreased in the MeDi group, including *Bifidobacterium* (*p* = 0.0002, FDR = 0.03), *Lachnospiraceae* NK4A136 group (*p* = 0.003, FDR = 0.09), *Turicibacter* (*p* = 0.001, FDR = 0.05), and a genus belonging to the *Lachnospiraceae* family (*p* = 0.0003, FDR = 0.09). Changes within the MeDi group and WD group from baseline to prior to behavior are displayed in Supplementary Figures S2-3.

### Cognitive function

**Figure 4.**
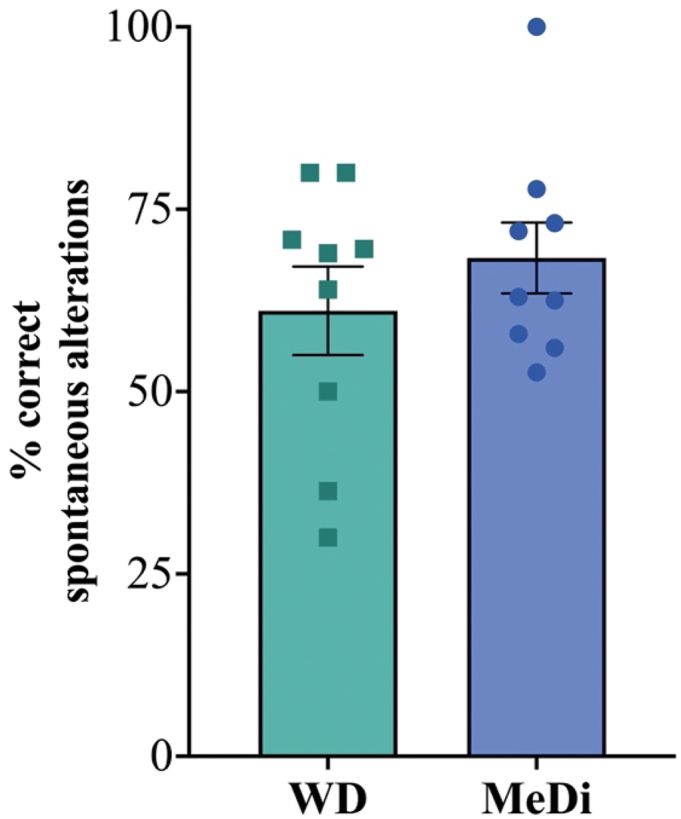
Percent correct alterations measured by the Y-maze did not differ between diets. A correct alteration score was calculated as the number of times the three arms were sequentially entered. The percent correct alterations are the number of correct alterations divided by the total number of alterations made. Data are represented as mean ± S.E.M. MeDi, Mediterranean diet (*n* = 9); WD, Western diet (*n* = 9).

A battery of behavioral assessments was used to assess the effect of diet on cognitive function after 14 weeks of diet. For the Y-Maze, there was no difference in percent correct spontaneous alterations between Diets (*p* = 0.37, Figure 4), suggesting that short-term recognition of novelty was not impacted by diet in these animals.

**Figure 5.**
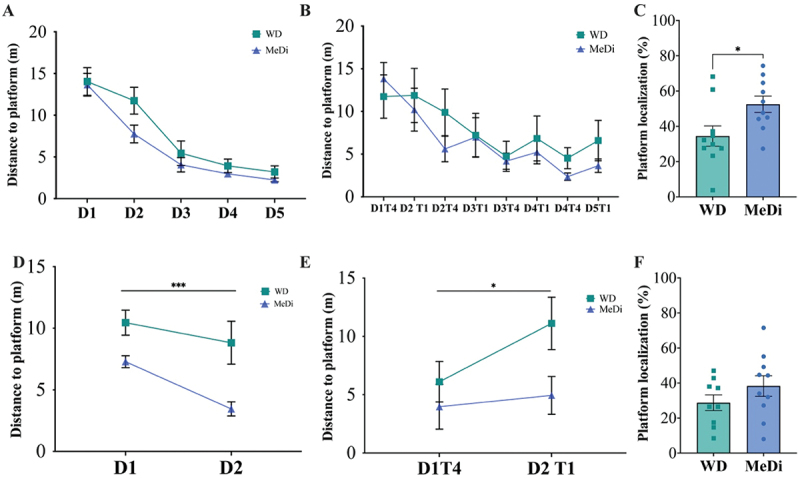
Morris water maze (MWM) behavioral assessment indicated better cognitive flexibility with the MeDi across a range of measures. MWM distance to platform (A), MWM overnight forgetting distance (B), and MWM platform quadrant localization (C; percent time in target – opposite quadrant). Reversal testing, a measurement of cognitive flexibility, was assessed by MWM distance to platform (D), MWM overnight forgetting distance (E), and MWM platform quadrant localization (F; percent time in target – opposite quadrant). Data are represented as mean ± S.E.M. MeDi, Mediterranean diet (*n* = 10); WD, Western diet (*n* = 9). *p = 0.04, **p = 0.006, ***p = 0.001.

For MWM, there was no difference between diet groups in distance swam to the platform or overnight forgetting (Figure 5A-B). For the 60 s probe trial at the end of testing, rats in the MeDi group localized the target platform quadrant better than the rats in the WD group (Figure 5C), suggesting that these rodents had better spatial reference memory than the WD group. For the 2-day reversal, MWM showed no interaction between Diet and Day. However, there were main effects of Diet (*p* = 0.001) and Day (*p* = 0.01) for distance to reach the platform. Regardless of the day, the rats in the MeDi group swam a shorter distance to reach the platform (WD group, 9.64 m vs MeDi Group, 4.27 m; difference, 5.37; Figure 5D), a result that is indicative of greater cognitive flexibility to learn new information. Further, overnight forgetting of newly learned information on the reversal was lower in the MeDi group indicating better retention of the previous day’s trial (WD group, 8.61 m vs MeDi Group, 4.46 m; difference, 4.16; Figure 5E). When testing localization of the reversal platform quadrant with a second probe trial at the end of testing, there was no difference between diets (Figure 5F). There was no difference between diet groups on swim speed during the MWM, 60 s probe trial, reversal, or reversal probe trial. Swim speed for the MWM is displayed in Supplementary Figure S4. Diet groups compared to chow diet MWM optimization animals are displayed in Supplementary Figure S5.

**Figure 6.**
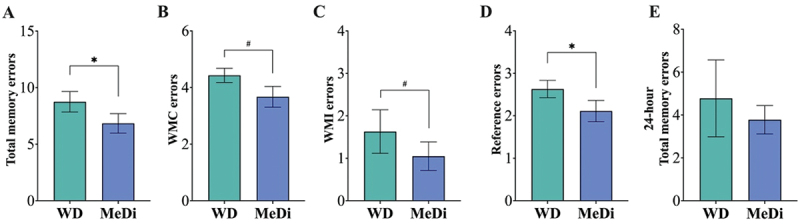
Water radial arm water (WRAM) maze behavioral assessments indicated better cognitive function with the Mediterranean diet (MeDi). Total (A), working memory correct (WMC; B), working memory incorrect (WMI; C), and reference memory (D) errors were, or had a trend to be, lower in the MeDi group. Total errors in the 24-hour delay (trials 3–4) of the WRAM did not differ between diet (E). Total memory error is the sum of WMC, WMI, and reference memory. Data are represented as mean ± S.E.M. MeDi, Mediterranean diet (*n* = 9); WD, Western diet (*n* = 9). #*p* < 0.1, *p < 0.05.

Following MWM, animals underwent WRAM testing for ten days (Figure 6A-E). One MeDi animal was not included in the analyses due to noncompliance; this animal repeatedly climbed out of the tank during trials, and this behavior persisted for the entirety of the assessment. Compared to the WD group, fewer total errors were committed by the MeDi group (*p* = 0.02). To discern specific domains of learning and memory, we assessed each error type on its own. There was a trend for the MeDi group to commit fewer WMC (*p* = 0.051) and WMI errors (*p* = 0.08). Reference memory errors were significantly lower with the MeDi group (*p* = 0.04). This is indicative of better working reference and spatial memory with the MeDi group compared to the WD. On day 11, after all animals had been trained on the task, a 24-hour delay was instilled between trials 2 and 3 to place a higher memory burden for trials 3 and 4 (Figure 6E). There were no differences in total memory errors between diet groups during the 24-hour delay (*p* = 0.60). Diet groups compared to chow diet WRAM optimization are displayed in Supplementary Figure S6.

### Peripheral immune function

A Multiplex was utilized to assess the effect of a MeDi compared to a WD on serum inflammatory markers. There was one outlier sample for the measurements of IL-13 utilizing the ROUT method and that was two standard deviations below the mean (mean = 625.9, standard deviation = 118.0, value = 332.3), therefore, that animal was not included in analyses (Supplementary Figure S7).

**Figure 7.**
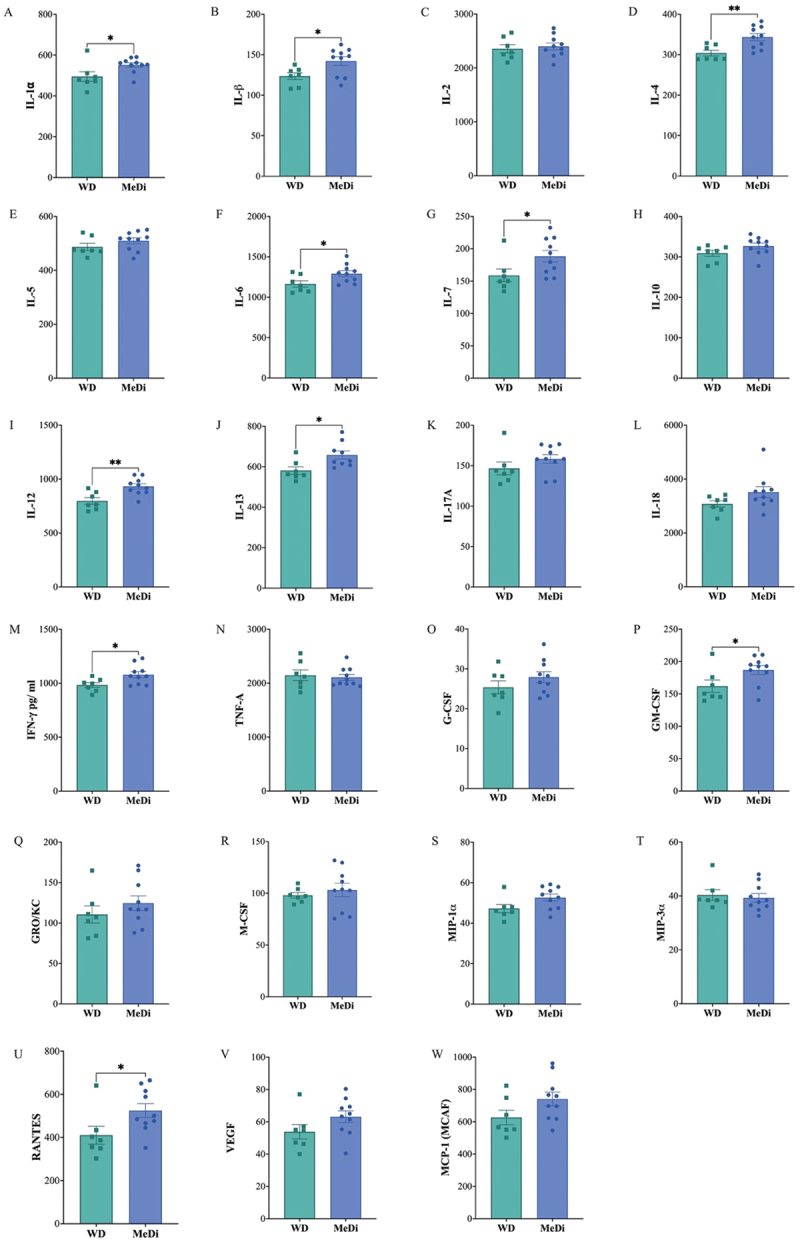
The Mediterranean diet (MeDi) had a higher concentration of individual pro-inflammatory and anti-inflammatory cytokines compared to the Western diet (WD). Cytokines assessed include (A-W): IL-1α, IL-1β, IL-2, IL-4, IL-5, IL-6, IL-7, IL-10, IL-12(p70), IL-13, IL-17α, IL-18, IFN-γ, tnf-α, G-CSF, GM-CSF, GRO/KC, M-CSF, MIP-1α, MIP-3α, RANTES, VEGF, and MCP-1. One sample in the MeDi was identified as an outlier using the ROUT method for IL-13. Therefore, the animal was excluded from this analysis. Serum was analyzed by a 23-multiplex. Data are represented as mean ± S.E.M. Units are pg/ml. MeDi, (*n* = 9); WD, (*n* = 7). *p < 0.05, **p < 0.01.

**Figure 8.**
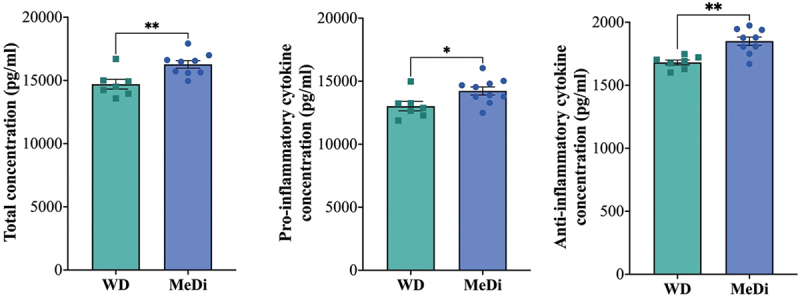
The Mediterranean diet (MeDi) had a higher concentration of total cytokines (A), pro-inflammatory cytokines (B), and anti-inflammatory cytokines (C) compared to the Western diet (WD) group. Pro-inflammatory cytokines include IL-1α, IL-1β, IL-2, IL-6, IL-7, IL-12(p70), IL-17α, IL-18, IFN-γ, TNF-α, GM-CSF, RANTES, MIP-1α, MIP-3α, MCP-1, G-CSF, GRO/KC, M-CSF, and VEGF. Anti-inflammatory cytokines include IL-4, IL-5, IL-10, and IL-13. Total concentration is the sum of anti- and pro-inflammatory cytokines measured in pg/ml. One sample in the MeDi group was identified as an outlier using the ROUT method for IL-13. Therefore, the animal was excluded from this analysis. MeDi, (*n* = 9); WD, (*n* = 7). *p < 0.05, **p < 0.01.

Compared to the rats in the WD group, the rats in the MeDi group had significantly higher concentrations (pg/ml) of 9 cytokines, including IL-1α, IL-1β, IL-4, IL-6, IL-7, IL-12 (p70), IL-13, GM-CSF, IFN-γ, and RANTES (*p* < 0.05, FDR < 0.1). There was a trend for MIP-1α to be higher in the MeDi group compared to the WD group (*p* < 0.09, FDR < 1.5). There were no differences in 11 cytokines, including IL-2, IL-5, IL-10, IL-17A, IL-18, G-CSF, GRO/KC, M-CSF, MIP-3α, TNF-α, VEGF, or MCP-1 between diet groups. Results are displayed in Figure 7, while means and FDR values are displayed in Supplementary Table S2. Overall, the MeDi group had a higher concentration of all cytokines compared to the WD group, as displayed in Figure 8A-C.

With both pro-inflammatory and anti-inflammatory cytokines increased in the MeDi group compared to the WD group, ratios of pro-/anti-inflammatory cytokines were assessed. Higher pro- to anti-inflammatory ratios have been associated with various states, including mild cognitive impairment, Alzheimer’s disease, and aging.^41–43^ After correcting for multiple comparisons, pro-/anti-inflammatory ratios did not differ by Diet (Supplementary Table 3). These data, together with the individual cytokine concentrations, suggest that the MeDi group did not exhibit an inflammatory state.

### Blood-brain barrier

**Figure 9.**
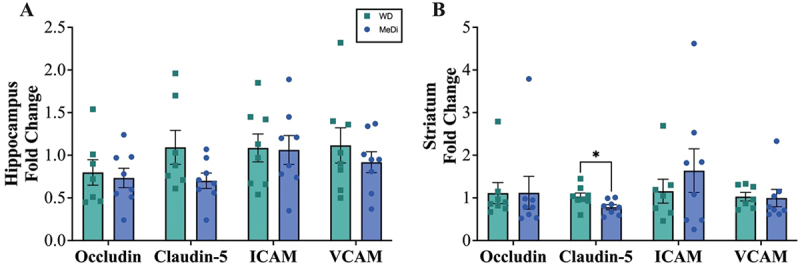
There were no differences in markers for blood-brain barrier (BBB) permeability in the hippocampus (A); however, claudin-5 in the striatum was higher in the Western diet (WD) group compared to the Mediterranean (MeDi) group (B). No other markers for BBB permeability were different between diets in the striatum. Markers were measured by qRT-pcr. Data are represented as mean ± S.E.M. ICAM, intercellular adhesion molecule-1; VCAM, vascular cell adhesion molecule 1. MeDi, (*n* = 7–8); WD, (*n* = 8);VCAM *p = 0.03.

To determine the difference between the MeDi and WD on markers of the blood-brain barrier, a potential mechanism influencing cognitive performance, we examined brain regions important for memory and learning, the hippocampus and striatum, by qRT-PCR. The blood-brain barrier (BBB) integrity was assessed by tight junction proteins, including Occludin and Claudin-5, and by immune cell infiltration, including ICAM and VCAM, indicating a leaky barrier. In the hippocampus, there were no differences in expression between diet groups for Occludin, Claudin-5, ICAM, or VCAM (Figure 9A). In the striatum, there was no difference in Occludin between diet groups, but Claudin-5 was decreased in rats in the MeDi group (*p* = 0.03; Figure 9B). However, there was no difference in ICAM or VCAM expression between diet groups (Figure 9B). Overall, hippocampal and striatum barrier integrity is maintained and not different between diet groups.

### Neuroinflammation

**Figure 10.**
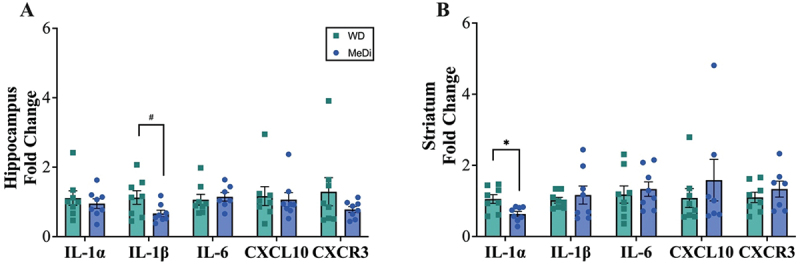
IL-1β gene expression had a trend to be higher in the Western diet (WD) group compared to the Mediterranean diet (MeDi) in the hippocampus; however, there were no differences for other cytokines or markers of cytokine recruitment (A). IL-1α gene expression was higher in the WD group compared to the MeDi group in the striatum (B); however, there were no differences for other cytokines or markers of cytokine recruitment. Markers were measured by qRT-pcr. Data are represented as mean ± S.E.M. CXCL10, C-X-C motif chemokine ligand 10; CXCR3, C-X-C motif chemokine receptor 3. MeDi, (*n* = 7–8); WD, (*n* = 8). *p = 0.01, #*p* = 0.054.

To determine differences between the MeDi and WD groups in immune function, gene expression of inflammatory cytokines that were increased in serum (IL-1α, IL-1β, and IL-6) and immune cell recruitment (CXCL10 and CXCR3) in the hippocampus and striatum were assessed (Figure 10A-B). In the hippocampus, there was a trend for a decrease in IL-1β gene expression in rats in the MeDi group compared to the rats in the WD group (*p* = 0.054). There was no difference between groups for expression of IL-1α, IL-6, CXCL10, or CXCR3. In the striatum, there was a decrease in IL-1α gene expression in the rats in the MeDi group compared to the rats in the WD group (*p* = 0.01). There was no difference between groups for expression of IL-1β, IL-6, CXCL10, or CXCR3.

### Astrocytes and microglial

**Figure 11.**
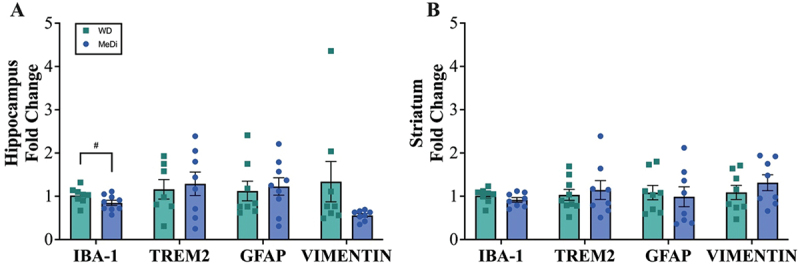
IBA-1 gene expression had a trend to be increased in the Western diet (WD) group compared to the Mediterranean (MeDi) group in the hippocampus; however, there were no differences for phagocytic activity or astrocytes (A). There was no difference between diet groups in the striatum for IBA-1, TREM2, GFAP, or vimentin (B). Markers were measured by qRT-pcr. Data are represented as mean ± S.E.M. IBA-1, ionized calcium-binding adaptor molecule 1; GFAP, glial fibrillary acidic protein; TREM2, triggering receptor expressed on myeloid cells 2. MeDi, (*n* = 8–9); WD, (*n* = 7–8). #*p* = 0.08.

To determine differences between the MeDi and WD in glial cell homeostasis, astrocytes were assessed by GFAP and vimentin gene expression, and microglia activity was assessed by IBA-1 and TREM2 gene expression in the hippocampus and striatum (Figure 11A-B). There was a trend for IBA-1 to have decreased gene expression in the hippocampus of rats in the MeDi group compared to the WD group (*p* = 0.08), but there was no difference in phagocytic activity measured by TREM2 between diet groups. GFAP and vimentin did not differ between diet groups. In the striatum, there was no difference in gene expression for GFAP, vimentin, IBA-1, or TREM2 between diet groups.

### Synaptic plasticity

**Figure 12.**
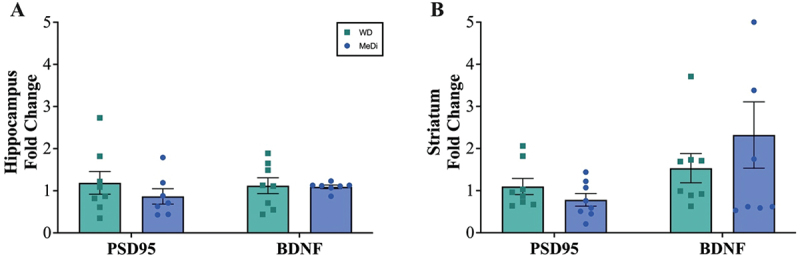
There was no difference in synaptic plasticity measured by PSD95 or BDNF in the hippocampus (A) or striatum (B). Markers were measured by qRT-pcr. Data are represented as mean ± S.E.M. BDNF, brain-derived neurotrophic factor; PSD95, postsynaptic density protein 95. MeDi, Mediterranean diet (*n* = 7–8); WD, Western diet (*n* = 8).

To determine differences between the MeDi and WD in synaptic plasticity, PSD95, and BDNF gene expression were measured in the hippocampus and striatum (Figure 12A-B). In both the hippocampus and striatum, there was no difference in gene expression of PSD95 and BDNF.

### Relationship between diet-modulated microbiota and outcomes

**Figure 13.**
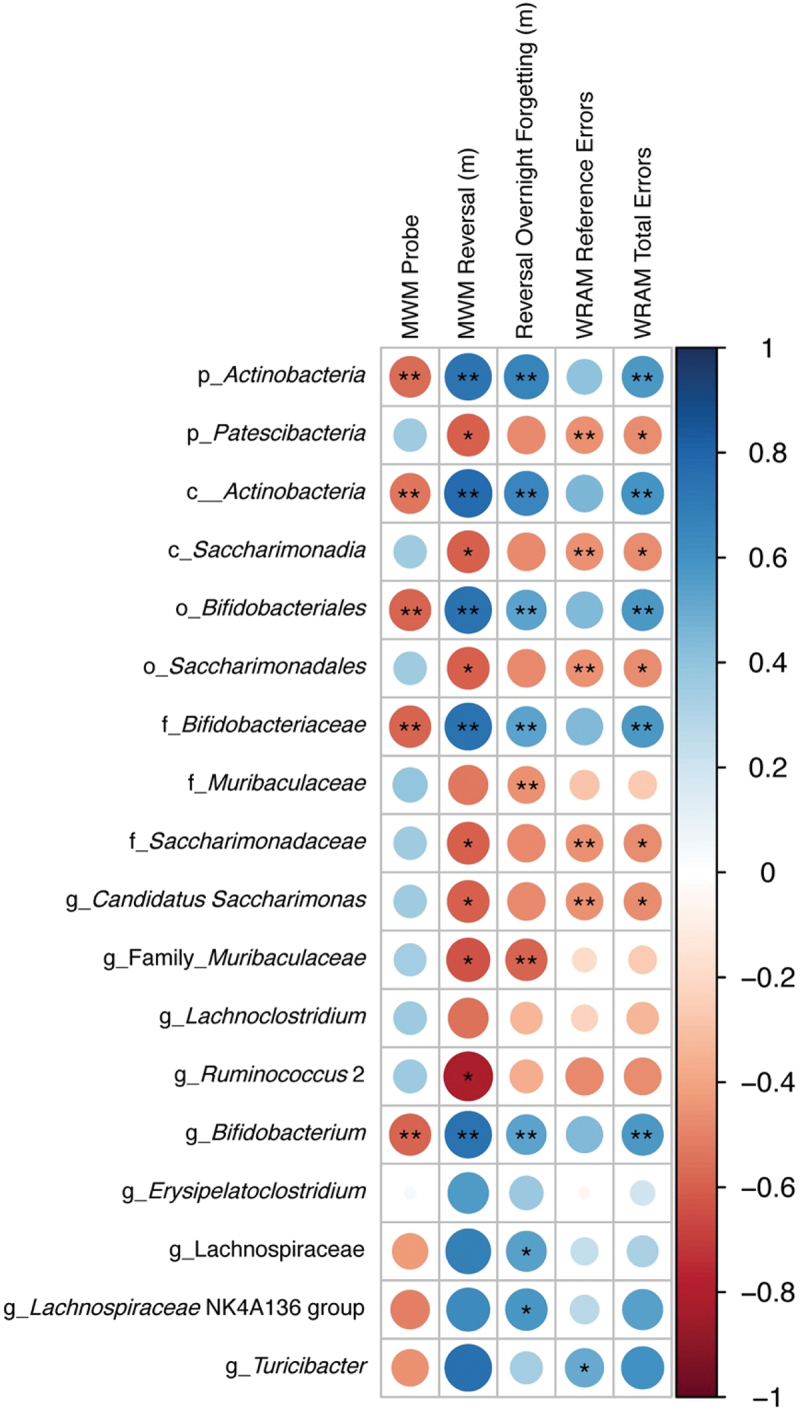
Diet-modulated gut microbiota was correlated with cognitive function. Heatmap of Pearson’s correlation analysis of behavioral assessments and microbiota. *R* values are indicated by the color key. Morris water maze, MWM; water radial arm maze, WRAM. *FDR < 0.1, **FDR < 0.05,

**Figure 14.**
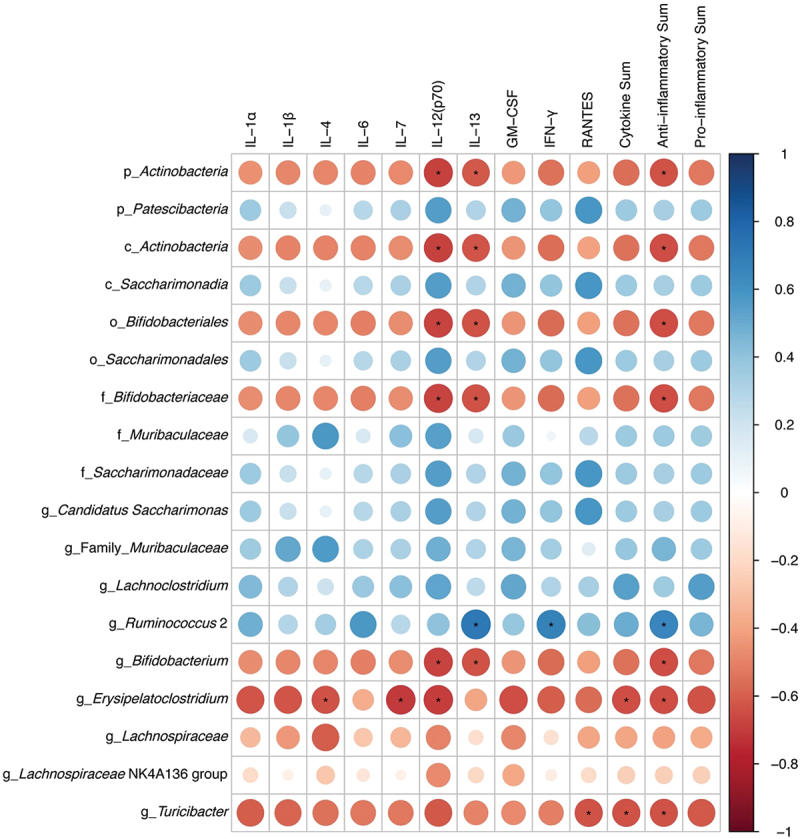
Diet-modulated gut microbiota was correlated with peripheral immune function. Heatmap of Pearson’s correlation analysis of microbiota and serum cytokine. *R* values are indicated by the color key. *FDR < 0.1,

To evaluate the impact of microbial changes on behavioral paradigms, we assessed the correlation between taxonomy, identified through LEfSe, and behavioral or serum cytokine outcomes between diets (Figs. 13–14).

For behavioral outcomes at the genus level (Figure 13), as the relative abundance of *Bifidobacterium* increased, cognitive performance was generally impaired. *Bifidobacterium* had a negative correlation with MWM platform quadrant localization and a positive correlation with MWM reversal distance, MWM reversal overnight forgetting, and total memory errors. *Candidatus Saccharimonas* had the opposite relationship. *Candidatus Saccharimonas* had a negative correlation with MWM reversal distance, reference memory errors, and total memory errors. A genus in the family of *Muribaculaceae* had a negative correlation with MWM reversal and MWM reversal overnight forgetting distance. *Ruminococcus* 2 relative abundance had a negative correlation with MWM reversal distance. *Turicibacter* relative abundance had a positive correlation with WRAM total errors. *Lachnoclostridium* and *Erysipelatoclostridium* did not correlate with any behavioral outcomes. This suggests that specific diet-modulated gut bacteria may correlate with types of spatial working and reference memory.

For serum cytokines and the genus level bacteria (Figure 14), *Bifidobacterium* had a negative correlation with IL-12, IL-13, and anti-inflammatory cytokine sum. *Ruminococcus* 2 abundance had a positive correlation with IL-13, IFN−γ, and anti-inflammatory cytokine sum. *Erysipelatoclostridium* abundance had the most correlations with serum cytokines; abundance was negatively correlated with IL-4, IL-7, IL-12, cytokine sum, and anti-inflammatory cytokine sum. *Turicibacter* relative abundance had a negative correlation with RANTES, cytokine sum, and anti-inflammatory cytokine sum. The abundance of *Candidatus Saccharimonas,* a genus in the family of *Muribaculaceae*, *Lachnoclostridium*, *Lachnospiracea*, and *Lachnospiracea* NK4A136 group did not correlate with any serum cytokine measurments.These findings support specific diet-modulated gut bacteria may correlate with anti- and pro-inflammatory cytokines.

## Discussion

The present study is the first to compare diets mimicking human consumption, the MeDi relative to the WD, on microbiota composition, cognitive function, serum lipids and cytokines, and gene expression in the brain in a translational rat model. We found that diet modulated two measures of β-diversity (Bray-Curtis dissimilarity and weighted UniFrac), but not unweighted Unifrac or α-diversity. At the genus level, the relative abundance of four bacteria increased in rats in the MeDi group, including *Candidatus Saccharimonas*, and five decreased, including *Bifidobacterium*. Compared to rats consuming the WD, rats in the MeDi group demonstrated cognitive flexibility and improvement in aspects of term spatial reference and working memory, as exhibited by the MWM platform localization and reversal testing and the WRAM. Further, rats in the MeDi group exhibited lower LDL cholesterol compared to the rats in the WD group. Peripheral immune function was modulated as specific pro- and anti-inflammatory cytokines in serum were higher in rats in the MeDi group; however, ratios of pro- to anti-inflammatory cytokines did not differ between diets. Changes in genera were correlated with specific behavioral outcomes and cytokine concentration. Analyses of the hippocampus and striatum demonstrated subtle region-specific changes in the expression of cytokine and BBB markers; however, there were no differences between diets for gene expression of glial cells or synaptic plasticity.

The MeDi, a neuroprotective diet, increases cognitive performance in youth populations.^8,44^ This is in stark contrast to a WD that promotes poor cognitive performance.^8,44^ Our data indicate that, compared to each other, the diets may have subtle effects on cognitive function in young adult male rats that were approximately equivalent to 18-year-old humans,^45^ as there was no effect of Diet on Y-maze recognition memory nor learning via MWM distance to platform. Previous research in mice demonstrated impairment in the Y-Maze; however, the use of a 60% high-fat diet for 18 weeks in that study constituted a higher percentage of fat compared to 43% and 35% fat in the MeDi and WD, respectively for 14 weeks, in our study.^46^ In another study, similar to our findings, young adult mice consuming a WD comparable in fat percentage for 12 weeks showed no difference in MWM distance to the platform compared to a low-fat control diet.^47^ This indicates the duration of time that animals are fed the diets may impact the results of certain cognitive assessments.

Intriguingly, when increasing cognitive demand during the reversal trial of the MWM and the WRAM, the rats in the MeDi group swam a shorter distance to the platform and committed fewer errors. As the age of the animals was equivalent to young adult humans at the time of cognitive assessments,^45^ by inference, dietary consumption throughout adolescence may play an important role in cognition, particularly for challenging tasks, in young adults. Cross-sectional studies indicate that the dietary intake in primary school and college students impacts cognitive performance, with fruit and vegetable intake associated with improved cognitive performance in contrast to fast food and soft drinks.^4,6^ Our findings support these clinical findings in a translational model.

The effect of the diet on cognitive function may be through dietary modulation of the gut microbiota as diet has the largest impact on gut microbiota composition.^13,14^ Similar to our results, in a study of 153 healthy individuals, α-diversity did not differ between those with a high MeDi adherence compared to low MeDi adherence, but microbiota composition was clustered by adherence (β-diversity).^3^ Fiber is the main and preferential energy substrate of microbes and intake can differ by dietary patterns. Fiber recommendations vary by country with a recommended intake of 25–35 grams/day (g/d, 25–32 g/d for women and 30–35 g/d for men) for most countries. However, global fiber intake is low and below recommendations with adult males consuming 15 to 25 g/d while females consume 14 to 21 g/d on average, with North America consuming less fiber than Europe.^48^ Further, protein and fat sources can also differentially impact the gut microbiota.^49–51^ Ultimately, as the MeDi and WD in our study differed in ingredient sources (i.e., olive oil vs butter), macronutrient ratio, and fiber quantity, changes to the gut microbiota and cognitive function may be attributed to an overall dietary pattern, including the individual components described above.

The relationship between microbiota and cognitive function has been examined mainly in the context of neurodegeneration, including Parkinson’s disease and Alzheimer’s disease.^52–55^ The gut microbiota of patients with cognitive impairment and Alzheimer’s disease differs from both healthy controls, indicating that there may be a shift in microbiota composition across the spectrum of brain function.^56^ *Bifidobacterium* abundance has been identified to change in both Alzheimer’s disease and Parkinson’s disease, with most studies reporting an increase in this genus.^52–54,56^ This study provides further evidence of the relationship between an overabundance of *Bifidobacterium* and cognitive health as increased abundance, as a result of the WD consumption, was associated with poorer cognitive performance. It is important to note that the genus *Bifidobacterium* has also been shown to have beneficial impacts on cognition and memory in preclinical and clinical studies.^57–61^ Whether a bacteria is beneficial or pathogenic is dependent on functionality and this can vary within species.^62^ For example, certain strains of *Escherichia coli* can be pathogenic, leading to diarrhea and urinary tract infections, while others have been used as a probiotic.^62^ Determining the functionality of species and strains within the *Bifidobacterium* genus will further our understanding of its relationship to cognitive health.

*Candidatus Saccharimonas* abundance was higher with the MeDi and associated with better cognitive performance. In translational models of obesity and Alzheimer’s disease, *Candidatus Saccharimonas* is found in lower abundances compared to wild-type animals.^63,64^ Our study further builds upon these findings and provides insight into the potential role of gut microbes in cognitive function.

There were increases in both pro-inflammatory and anti-inflammatory cytokines with no changes to pro- to anti-inflammatory ratios in rats in the MeDi group compared to rats in the WD group. This indicates that the MeDi resulted in an increase in overall cytokine concentration. Immune function can be modulated by the intake of omega-6 and omega-3 fatty acids with a higher ratio of omega-6: omega-3 associated with lower immune cell function and cytokine expression.^65,66^ In this study, the rats in MeDi group contained a lower omega-6: omega-3 ratio compared to the rats in the WD group. These differences in fatty acid concentration between diet compositions are expected as the diets mimic human consumption. The higher level of cytokines in rats in the MeDi group compared to the WD group may indicate that the MeDi group has an active immune response, while the WD group may be suppressed in part due to the omega-6: omega-3 ratio.

Increased omega-6: omega-3 ratios have been associated with increased LDL cholesterol.^67^ Our study replicates these findings in our model as serum LDL cholesterol concentration was increased in rats consuming the WD compared to the MeDi. LDL cholesterol levels have been associated with cognitive decline and dementia risk.^68,69^ Although animals in this study were young and were not subject to the long-term effects of increased LDL cholesterol such as atherosclerosis and reduced blood flow, risk factors for dementia, WD-consuming animals may have been subject to the start of this process resulting in the subtle differences in cognitive performance between diets. Interestingly, gut microbiota can regulate cholesterol absorption and plasma cholesterol levels.^70^ Two bacteria in our study identified by LEfSe analysis to be increased in WD-consuming rats, *B. pseudolongum*, and *Lachnospiraceae*, have been identified to be highly interactive with diet-derived cholesterol or associated with increased LDL cholesterol.^71,72^ This indicates a potential target for intervention to mitigate the risk of cognitive deficits by decreasing the modifiable risk factor of LDL through gut microbiota modulation.

Although peripheral changes in immune function and LDL concentrations were exhibited in serum, subtle or no changes for BBB markers, neuroinflammation, gliosis, or synaptic plasticity were observed in brain regions important for memory and learning. This may be in part due to time and region-dependent changes in immune response to a high-fat diet.^73–76^ Short-term diets, ranging from ≤1 week to 4 weeks, have demonstrated substantial differences in neuroinflammatory markers, indicating a rapid diet-induced response,^74,75^ whereas neuroinflammation did not differ with long-term diets of 16–20 weeks.^73,76^ Time-dependent changes are not restricted to neuroinflammation as BBB integrity,^74,77^ markers of synaptic plasticity,^78^ and gliosis^78,79^ can differ by diet duration. Interestingly, markers of astrocyte expression have exhibited increased expression at weeks 1 and 2 but not at week 3, with a return in increased expression at a long-term diet duration of 8 months, indicating a period effect.^80^ Together, this indicates changes in response to dietary manipulation are temporal; therefore, our diet duration may not have captured short- or long-term negative effects.

The lack of a chow control diet did not allow for its comparison to the MeDi and WD, however, this study aimed to compare the nutrient- and fiber-dense diet, MeDi, compared to the commonly consumed WD, therefore creating a more translatable pre-clinical dietary study intervention. The WD was utilized in this study as the control as it is the standard/most commonly adhered-to diet in America. Clinical dietary interventions are formulated with interventions such as the MeDi to mitigate the negative health outcomes associated with the WD. In pre-clinical models, the lack of a standard rodent chow is not uncommon. Standard rodent chow is variable across commercial formulations, resulting in differing gut microbiota compositions, thus limiting reproducibility between researchers and translatability to humans.^81–83^ In place of a standard rodent chow, research studies have compared high-fat and low-fat diets to each other.^47,84–87^ The gut-brain axis mechanisms were explored in transgenic mice consuming a modified Mediterranean-style diet compared to a WD to mimic human consumption.^22^ Non-human primate models mimic human consumption by comparing the MeDi to the WD.^21,23,24,88^ This indicates the growing usage of the more commonly followed human diets as the control in studies.

There are a few limitations in this study. The first is in the applicability of the study, with the use of male rats only. As female rats depleted of endogenous circulating hormones develop cognitive impairment and gut dysbiosis,^89,90^ it will be important to explore the effect of sex in future study iterations. Second, the unforeseen power outages may have caused undue stress to animals included in this study; however, we attempted to mitigate these effects by delaying study activities for at least two weeks, and the effects were equal for both MeDi and WD fed animals. Third, as serum and tissues were collected from post-behavior assessments, duration and behavioral assessments may have impacted outcomes. Future studies may consider a subgroup of animals whose samples are collected prior to an additional group undergoing neurobehavior assessments. However, this would not allow for correlations between behavior and outcomes. Finally, due to the observational nature of this study of diet-modulated gut microbiota and cognitive function, future studies are needed to determine the direct impact of these identified microbes on cognitive performance.

This study is unique in that it is the first to assess the effects of the MeDi on microbiota and cognitive function outcomes in rats relative to the WD. Our diets were modeled on human consumption^20^ and the variety of ingredients utilized mimicked the complexity of human diets. Further, various studies investigating the impact of fat on cognitive or microbial changes contained only insoluble fiber, such as cellulose, or did not list their fiber content.^46,47,91,92^ The fiber sources are important as the absence of soluble fiber results in the loss of microbial species and drives diet-induced obesity.^93,94^ By providing inulin in both the MeDi and WD based on dietary records,^20^ the diets used in this study emulate those in humans and hence our microbiota and cognitive function data are translatable.

## Conclusion

In summary, we found that the MeDi group had better cognitive function compared to the WD group. Diets modulated gut microbiota at all taxonomic levels and these changes were associated with learning and memory. The diets impacted peripheral immune function as the MeDi group had a higher concentration of both anti- and pro-inflammatory cytokines compared to the WD. However, brain markers for neuroinflammation, BBB, glial cells, and synaptic plasticity were generally unchanged. Our results suggest that there may be a relationship between diet-modulated microbiota, peripheral immune function, and cognitive function. Additional studies are needed to determine the causality between diet-modulated gut microbiota, immune function, and cognitive function, and to explore additional brain mechanisms.

## Abbreviations

BBB: blood-brain barrier
BDNF: brain-derived neurotrophic factor
CXCL10: C-X-C motif chemokine 10
CXCR3: C-X-C motif chemokine receptor 3
FDR: false discovery rate
GFAP: glial fibrillary acidic protein
g/d: Grams/Day
HDL: high-density lipoprotein
HPRT1: hypoxanthine phosphoribosyltransferase 1
IBA-1: ionized calcium-binding adaptor molecule 1
ICAM: intercellular adhesion molecule 1
LDL: low-density lipoprotein
LEfSe: linear discriminant analysis (LDA) effect size
MeDi: Mediterranean diet
MWM: Morris water maze
PSD95: postsynaptic density protein-95
TREM2: triggering receptor expressed on myeloid cells 2
WD: Western diet
WMC: working memory correct
WMI: working memory incorrect
WRAM: water radial arm maze

## Supplementary Material

MeDi_Supplementary_Material_8.6.24.docx

## Data Availability

The data that support the findings of this study are openly available in National Center for Biotechnology Information (NCBI)-Sequence Read Archive (SRA) at https://www.ncbi.nlm.nih.gov/sra, reference number PRJNA934338 (16S rRNA sequencing dataset).
