# Supplementary material for: Comparison between two divergent diets, Mediterranean and Western, on gut microbiota and cognitive function in young sprague dawley rats
Source: Gut Microbes Rep. 2024 Dec 18;1(1):2439490. doi: 10.1080/29933935.2024.2439490 (PMC11800364; doi:10.1080/29933935.2024.2439490)
Supplement: MeDi_Supplementary_Material_8.6.24.docx [file KGMR_A_2439490_SM7744.docx]

Supplementary Material

Comparison Between Two Divergent Diets, Mediterranean and Western, on Gut Microbiota and Cognitive Function in Young Sprague Dawley Rats.

Rebecca J. Solch-Ottaiano,^1,2^ Elizabeth B. Engler-Chiurazzi,^2,3^ Colin Harper,^1,2^ Savannah Wasson,^1^ Sharon Ogbonna,^1^ Blake Ouvrier,^2,3^ Hanyun Wang,^3^ Madison Prats,^1,2^ Katherine McDonald,^3^ Ifechukwude J. Biose,^2,3^ Lori A. Rowe,^4^ MaryJane Jones,^5^ Chad Steele,^5^ Gregory Bix,^2,3^ Demetrius M. Maraganore^1,2^*

^1^ Clinical Neuroscience Research Center, Department of Neurology, Tulane University School of Medicine, New Orleans, LA, USA

^2^Tulane Brain Institute, Tulane University, New Orleans, LA, USA

^3^Clinical Neuroscience Research Center, Department of Neurosurgery, Tulane University School of Medicine, New Orleans, LA, USA

^4^Virus Characterization, Isolation, Production and Sequencing Core, Department of Microbiology, Tulane National Primate Center, Covington, LA, USA

^5^Department of Microbiology and Immunology, Tulane University School of Medicine, New Orleans, LA, USA

***** Correspondence: Demetrius M. Maraganore, MD, FAAN, dmaraganore@tulane.edu

## Supplementary Figures

**Supplementary Figure 1.** Relative abundance of Actinobacteria with (**A**) and without (**B**) the identified outlier. This outlier was confirmed using the ROUT method on the relative abundance of Actinobacteria. Additionally, the value was two standard deviations above the mean (mean=0.103, standard deviation=0.13, value=0.616). Data are represented as mean ± standard deviation.


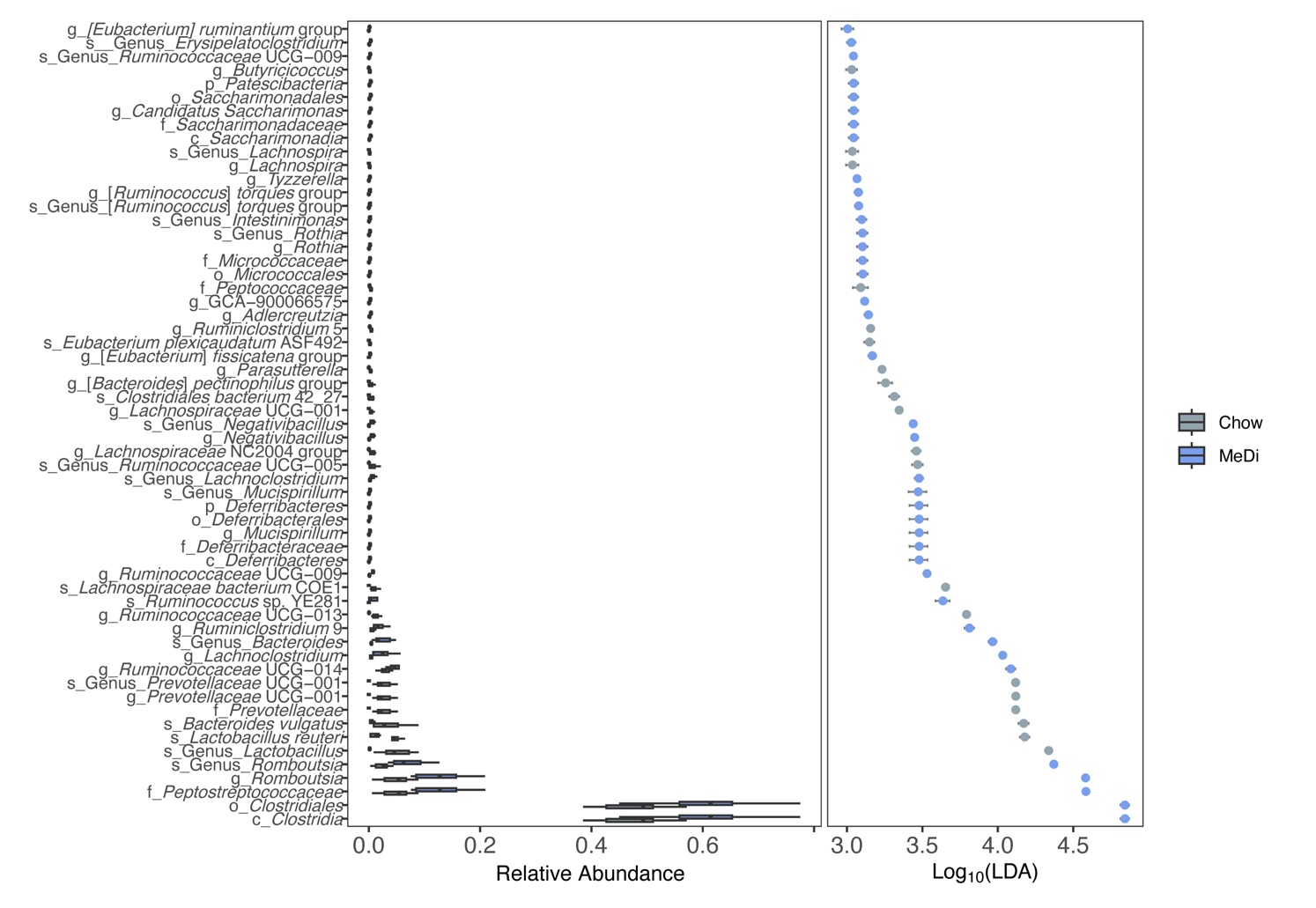


**Supplementary Figure 2.** The MeDi induced gut microbial changes across taxonomic levels compared to a chow diet (baseline). Relative abundance and linear discriminant analysis values determined via Linear discriminant analysis Effect Size. Relative abundance is represented as interquartile range. MeDi, Mediterranean diet (n=10)


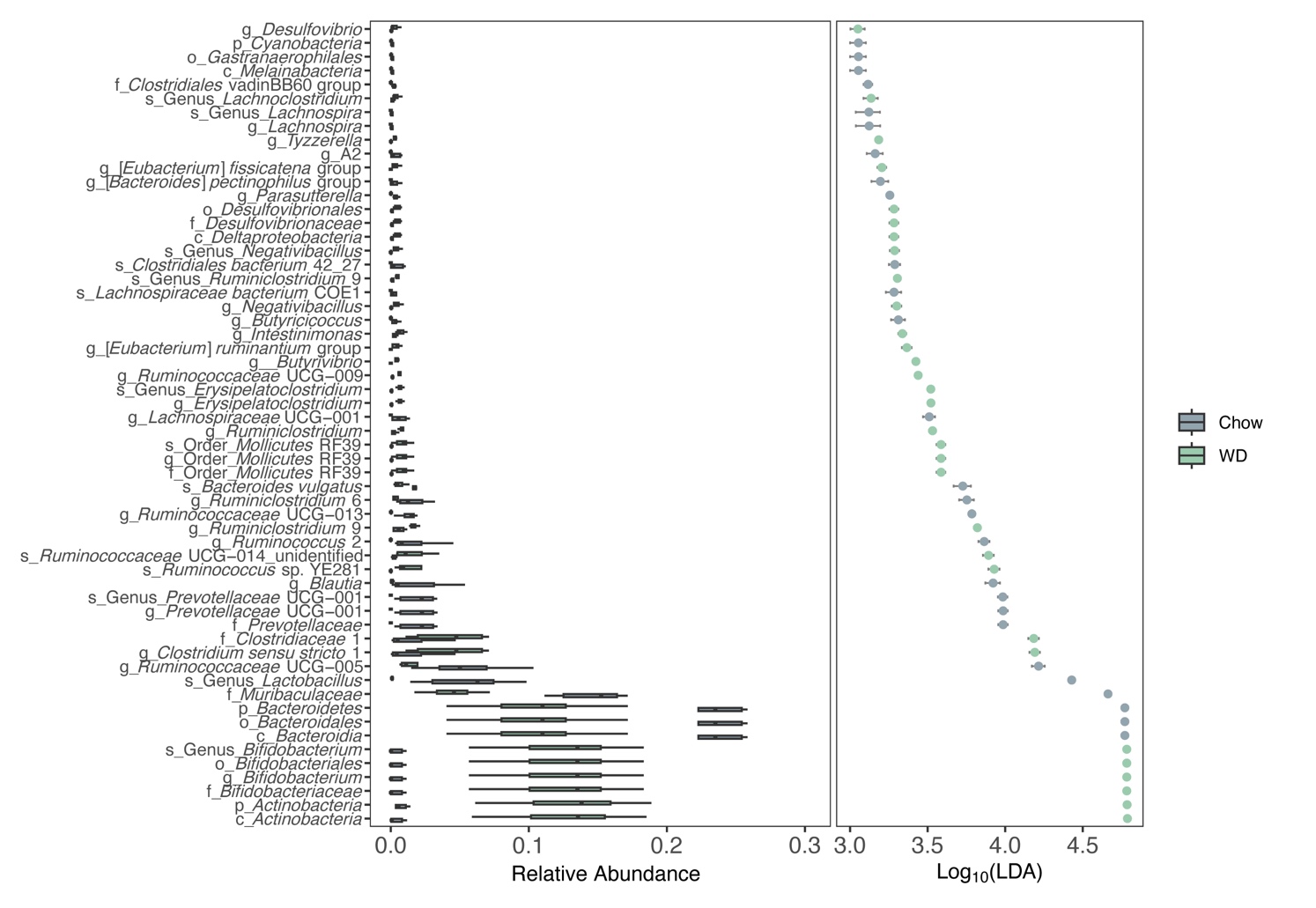


**Supplementary Figure 3.** The WD-induced gut microbial changes across taxonomic levels compared to a chow diet (baseline). Relative abundance and linear discriminant analysis values determined via Linear discriminant analysis Effect Size. Relative abundance is represented as interquartile range. WD, Western diet (n=9)

**Supplementary Figure 4.** Morris water maze (MWM) speed (m/s) for MeDi and WD groups did not differ between diets. Data are represented as mean ± S.E.M. MeDi, Mediterranean diet (n=10); WD, Western diet (n=9).

**Supplementary Figure 5.** Morris water maze (MWM) latency (s) for MeDi and WD groups compared to chow optimization animals (9-week-old Sprague Dawley rats). Distance was not recorded by AnyMaze: therefore, latency to the platform is displayed. Data are represented as mean ± S.E.M. MeDi, Mediterranean diet (n=10); WD, Western diet (n=9); Chow (n=6).

**Supplementary Figure 6.** Total errors in the Water radial arm maze for MeDi and WD groups compared to chow optimization animals (11-week-old Sprague Dawley rats) across days (**A**) and by Diet (**B**). Total error is the sum of working memory correct, working memory incorrect, and reference memory. Data are represented as mean ± S.E.M. MeDi, Mediterranean diet (n=10); WD, Western diet (n=9); Chow (n=6). *p<0.05.

**Supplementary Figure 7.** Concentration of serum IL-13 with (**A**) and without (**B**) the identified outlier. This outlier was confirmed using the ROUT method on the IL-13 concentration. Additionally, the value was two standard deviations below the mean (mean=625.9, standard deviation=118.0, value=332.3). Data are represented as mean ± standard deviation.

**Supplementary Table 1.** Linear discriminant analysis Effect Size of Mediterranean and Western Diet microbiota.

|  | Diet^1^ | LDA upper | LDA mean | LDA lower | P value | FDR^2^ |
| --- | --- | --- | --- | --- | --- | --- |
| **Phylum** |  |  |  |  |  |  |
| Actinobacteria | WD | 4.78 | 4.77 | 4.76 | 0.0002 | 0.03 |
| Patescibacteria | MeDi | 3.14 | 3.12 | 3.09 | 0.003 | 0.08 |
| **Class** |  |  |  |  |  |  |
| Actinobacteria | WD | 4.79 | 4.78 | 4.77 | 0.0002 | 0.03 |
| Saccharimonadia | MeDi | 3.14 | 3.12 | 3.09 | 0.003 | 0.08 |
| **Order** |  |  |  |  |  |  |
| Bifidobacteriales | WD | 4.80 | 4.79 | 4.77 | 0.0002 | 0.03 |
| Saccharimonadales | MeDi | 3.14 | 3.12 | 3.09 | 0.003 | 0.08 |
| **Family** |  |  |  |  |  |  |
| Bifidobacteriaceae | WD | 4.80 | 4.79 | 4.77 | 0.0002 | 0.03 |
| Muribaculaceae | MeDi | 4.36 | 4.34 | 4.31 | 0.001 | 0.07 |
| Saccharimonadaceae | MeDi | 3.14 | 3.12 | 3.09 | 0.003 | 0.08 |
| **Genus** |  |  |  |  |  |  |
| *Bifidobacterium* | WD | 4.80 | 4.79 | 4.77 | 0.0002 | 0.03 |
| *Candidatus Saccharimonas* | MeDi | 3.14 | 3.12 | 3.09 | 0.003 | 0.08 |
| *Lachnoclostridium* | MeDi | 4.05 | 4.03 | 4.00 | 0.003 | 0.08 |
| Family_*Lachnospiraceae* | WD | 4.60 | 4.58 | 4.55 | 0.0003 | 0.03 |
| *Lachnospiraceae NK4A136 group* | WD | 4.05 | 4.02 | 3.98 | 0.003 | 0.09 |
| *Muribaculaceae* | MeDi | 4.36 | 4.33 | 4.31 | 0.001 | 0.07 |
| *Ruminococcaceae* | MeDi | 3.47 | 3.45 | 3.42 | 0.001 | 0.04 |
| *Turicibacter* | WD | 4.13 | 4.10 | 4.07 | 0.001 | 0.05 |
| **Species** |  |  |  |  |  |  |
| *B. pseudolongum* | WD | 4.8 | 4.8 | 4.8 | 0.0002 | 0.03 |
| NK4A214_group_ unidentified | WD | 3.1 | 3.1 | 3.1 | 0.0002 | 0.03 |
| s__Family_ Lachnospiraceae | WD | 4.6 | 4.6 | 4.6 | 0.0003 | 0.03 |
| s__Genus_ Muribaculaceae | MeDi | 4.0 | 4.0 | 4.0 | 0.0003 | 0.03 |
| s__un_g__ Ruminococcaceae | MeDi | 3.5 | 3.4 | 3.4 | 0.001 | 0.04 |
| s__un_g__ Turicibacter | WD | 4.1 | 4.1 | 4.1 | 0.001 | 0.05 |
| s__Genus_ UCG-005 | WD | 3.7 | 3.6 | 3.5 | 0.001 | 0.07 |
| s__un_g__ Candidatus_Saccharimonas | MeDi | 3.1 | 3.1 | 3.1 | 0.003 | 0.08 |
| s__un_g__ Lachnospiraceae_NK4A136_group | WD | 4.0 | 3.9 | 3.9 | 0.003 | 0.08 |
| s__Genus_ Lachnoclostridium | MeDi | 4.0 | 4.0 | 4.0 | 0.003 | 0.09 |

^1^Diet in which the relative abundance was higher.

^2^Threshold of p<0.1.

FDR, false discovery rate; LDA, least discriminant analysis. Mediterranean diet (n=9-10); WD, Western diet (n=9).

**Supplementary Table 2.** Mediterranean and Western Diet Serum Cytokines.

| Cytokine (pg/mL) | WD ± SEM | MeDi ± SEM | P value^1^ | FDR P value^2^ |
| --- | --- | --- | --- | --- |
| IL-1⍺ | 594.8 ± 23.88 | 551.2 ± 11.48 | **0.03** | **0.093** |
| IL-1β | 123.5 ± 4.24 | 142.2 ± 5.48 | **0.02** | **0.093** |
| IL-2 | 2355 ± 77.05 | 2400 ± 63.71 | 0.66 | 0.727 |
| IL-4 | 304 ± 7.03 | 343.5 ± 8.84 | **0.01** | **0.093** |
| IL-5 | 486.8 ± 13.02 | 509.6 ± 11.21 | 0.21 | 0.301 |
| IL-6 | 1164 ± 39.21 | 1292 ± 36.23 | **0.03** | **0.093** |
| IL-7 | 158.9 ± 9.74 | 188.4 ± 8.89 | **0.04** | **0.093** |
| IL-10 | 309.1 ± 7.57 | 326.7 ± 7.1 | 0.12 | 0.186 |
| IL-12 | 796.7 ± 30.79 | 932 ± 24.64 | **0.004** | **0.093** |
| IL-13^3^ | 581.3 ± 18.19 | 658.5 ± 20.24 | **0.02** | **0.093** |
| IL-17A | 146.7 ± 7.86 | 158.3 ± 5.34 | 0.22 | 0.301 |
| IL-18 | 3078 ± 118.3 | 3513 ± 203.7 | 0.12 | 0.186 |
| G-CSF | 25.37 ± 1.63 | 27.93 ± 1.36 | 0.25 | 0.323 |
| GM-CSF | 161.9 ± 9.59 | 187 ± 6.87 | **0.04** | **0.093** |
| GRO/KC | 110.7 ± 10.64 | 124.6 ± 8.98 | 0.33 | 0.403 |
| INF-γ | 983.1 ± 22.93 | 1079 ± 28.24 | **0.03** | **0.093** |
| M-CSF | 98.1 ± 2.64 | 103.2 ± 6.43 | 0.53 | 0.616 |
| MIP-1⍺ | 47.23 ± 2.01 | 52.66 ± 1.73 | *0.06* | 0.127 |
| MIP-3⍺ | 40.36 ± 1.97 | 39.32 ± 1.58 | 0.69 | 0.727 |
| RANTES | 410.4 ± 41.59 | 524.5 ± 31.97 | **0.04** | **0.093** |
| TNF-⍺ | 2147 ± 97.63 | 2109 ± 54.9 | 0.72 | 0.727 |
| VEGF | 53.86 ± 4.47 | 63.14 ± 3.61 | 0.12 | 0.186 |
| MCP-1 (MCAF) | 626.5 ± 44.7 | 740.5 ± 42.51 | *0.09* | 0.174 |

^1^Data analyzed via unpaired Student’s t-test. Bold p values indicate p<0.05; italicized p values represent p<0.1.

^2^False discovery rate p value. Bold p values indicate p<0.1.

^3^One sample in the MeDi was identified as an outlier using the ROUT method. Value = 332.32. The mean of MeDi IL-13 with the outlier is 625.9 (± 37.31) and the p-value with the outlier included is 0.36.

FDR, false discovery rate; SEM, standard error of mean. MeDi, Mediterranean diet (n=10/group); WD, Western Diet (n=7/group).

**Supplementary Table 3.** Ratios of pro/anti-inflammatory cytokines for Mediterranean and Western Diet.

| Cytokine (pg/mL) | WD mean ± SEM | MeDi mean ± SEM | P value^1^ | FDR P value^2^ |
| --- | --- | --- | --- | --- |
| IL-1α/IL-4 | 1.627 ± 0.063 | 1.609 ± 0.036 | 0.804 | 0.907 |
| IL-1β/IL-4 | 0.406 ± 0.011 | 0.413 ± 0.012 | 0.677 | 0.838 |
| IL-2/IL-4 | 7.757 ± 0.237 | 7.007 ± 0.182 | 0.022 | 0.397 |
| IL-6/IL-4 | 3.846 ± 0.169 | 3.772 ± 0.105 | 0.698 | 0.838 |
| IL-7/ IL-4 | 0.522 ± 0.027 | 0.548 ± 0.02 | 0.455 | 0.817 |
| IL-12(p70)/IL-4 | 2.628 ± 0.112 | 2.721 ± 0.074 | 0.475 | 0.817 |
| IL-17/IL-4 | 0.482 ± 0.02 | 0.462 ± 0.014 | 0.402 | 0.811 |
| IL-18/IL-4 | 10.154 ± 0.432 | 10.249 ± 0.544 | 0.901 | 0.977 |
| G-CSF/IL-4 | 0.084 ± 0.006 | 0.081 ± 0.003 | 0.697 | 0.838 |
| GM-CSF/ IL-4 | 0.532 ± 0.026 | 0.545 ± 0.018 | 0.670 | 0.838 |
| GRO/ KC / IL-4 | 0.362 ± 0.03 | 0.361 ± 0.022 | 0.977 | 0.989 |
| IFNγ/IL-4 | 3.241 ± 0.092 | 3.152 ± 0.086 | 0.499 | 0.833 |
| M-CSF/ IL-4 | 0.323 ± 0.01 | 0.301 ± 0.018 | 0.346 | 0.735 |
| MIP-1α/IL-4 | 0.155 ± 0.005 | 0.153 ± 0.003 | 0.734 | 0.854 |
| MIP-3Α/ IL-4 | 0.133 ± 0.006 | 0.115 ± 0.004 | **0.017** | 0.397 |
| RANTES/ IL-4 | 1.34 ± 0.109 | 1.538 ± 0.102 | 0.213 | 0.628 |
| TNFα/IL-4 | 7.085 ± 0.355 | 6.163 ± 0.189 | **0.025** | 0.397 |
| VEGF/ IL-4 | 0.176 ± 0.011 | 0.184 ± 0.009 | 0.612 | 0.836 |
| MCP-1/ IL-4 | 2.052 ± 0.111 | 2.158 ± 0.116 | 0.535 | 0.833 |
| IL-1α/IL-5 | 1.016 ± 0.039 | 1.083 ± 0.016 | 0.092 | 0.470 |
| IL-1β/IL-5 | 0.254 ± 0.009 | 0.278 ± 0.006 | **0.033** | 0.397 |
| IL-2/IL-5 | 4.852 ± 0.175 | 4.716 ± 0.1 | 0.479 | 0.817 |
| IL-6/IL-5 | 2.409 ± 0.126 | 2.54 ± 0.066 | 0.332 | 0.735 |
| IL-7/ IL-5 | 0.326 ± 0.015 | 0.369 ± 0.012 | **0.045** | 0.397 |
| IL-12(p70)/IL-5 | 1.64 ± 0.059 | 1.829 ± 0.028 | **0.006** | 0.397 |
| IL-17A/IL-5 | 0.301 ± 0.011 | 0.31 ± 0.007 | 0.459 | 0.817 |
| IL-18/IL-5 | 6.35 ± 0.287 | 6.912 ± 0.395 | 0.307 | 0.735 |
| G-CSF/IL-5 | 0.053 ± 0.004 | 0.055 ± 0.002 | 0.628 | 0.836 |
| GM-CSF/ IL-5 | 0.332 ± 0.015 | 0.366 ± 0.007 | 0.046 | 0.397 |
| GRO/ KC / IL-5 | 0.226 ± 0.018 | 0.243 ± 0.013 | 0.456 | 0.817 |
| IFNγ/IL-5 | 2.025 ± 0.053 | 2.121 ± 0.048 | 0.201 | 0.628 |
| M-CSF/ IL-5 | 0.202 ± 0.005 | 0.203 ± 0.012 | 0.955 | 0.989 |
| MIP-1α/IL-5 | 0.097 ± 0.003 | 0.103 ± 0.002 | 0.129 | 0.498 |
| MIP-3Α/ IL-5 | 0.083 ± 0.003 | 0.077 ± 0.003 | 0.189 | 0.628 |
| RANTES/ IL-5 | 0.836 ± 0.065 | 1.03 ± 0.059 | **0.047** | 0.397 |
| TNFα/IL-5 | 4.421 ± 0.2 | 4.155 ± 0.136 | 0.271 | 0.717 |
| VEGF/ IL-5 | 0.11 ± 0.007 | 0.123 ± 0.005 | 0.130 | 0.498 |
| MCP-1/ IL-5 | 1.28 ± 0.061 | 1.448 ± 0.063 | 0.087 | 0.470 |
| IL-1α/IL-10 | 1.6 ± 0.059 | 1.691 ± 0.038 | 0.191 | 0.628 |
| IL-1β/IL-10 | 0.4 ± 0.012 | 0.435 ± 0.013 | 0.083 | 0.470 |
| IL-2/IL-10 | 7.623 ± 0.182 | 7.369 ± 0.216 | 0.412 | 0.811 |
| IL-6/IL-10 | 3.773 ± 0.118 | 3.966 ± 0.118 | 0.280 | 0.717 |
| IL-7/ IL-10 | 0.514 ± 0.027 | 0.577 ± 0.025 | 0.113 | 0.481 |
| IL-12(p70)/IL-10 | 2.589 ± 0.126 | 2.858 ± 0.07 | 0.063 | 0.455 |
| IL-17/IL-10 | 0.474 ± 0.021 | 0.484 ± 0.012 | 0.668 | 0.838 |
| IL-18/IL-10 | 9.97 ± 0.367 | 10.81 ± 0.683 | 0.354 | 0.735 |
| G-CSF/ IL-10 | 0.082 ± 0.004 | 0.086 ± 0.004 | 0.543 | 0.833 |
| GM-CSF/ IL-10 | 0.523 ± 0.026 | 0.572 ± 0.016 | 0.111 | 0.481 |
| GRO/KC / IL-10 | 0.358 ± 0.033 | 0.381 ± 0.025 | 0.584 | 0.836 |
| IFNγ/IL-10 | 3.189 ± 0.087 | 3.311 ± 0.084 | 0.341 | 0.735 |
| M-CSF / IL-10 | 0.319 ± 0.012 | 0.317 ± 0.02 | 0.950 | 0.989 |
| MIP-1α | 0.153 ± 0.005 | 0.162 ± 0.005 | 0.276 | 0.717 |
| MIP-3Α/ IL-10 | 0.131 ± 0.006 | 0.12 ± 0.004 | 0.160 | 0.585 |
| RANTES/ IL-10 | 1.327 ± 0.123 | 1.611 ± 0.103 | 0.098 | 0.470 |
| TNFα/IL-10 | 6.961 ± 0.312 | 6.481 ± 0.217 | 0.212 | 0.628 |
| VEGF/ IL-10 | 0.175 ± 0.015 | 0.193 ± 0.009 | 0.297 | 0.735 |
| MCP-1/ IL-10 | 2.032 ± 0.15 | 2.267 ± 0.118 | 0.232 | 0.660 |
| IL-1α/IL-13^3^ | 0.858 ± 0.056 | 0.856 ± 0.021 | 0.980 | 0.989 |
| IL-1β/IL-13^3^ | 0.213 ± 0.009 | 0.221 ± 0.011 | 0.617 | 0.836 |
| IL-2/IL-13^3^ | 4.078 ± 0.2 | 3.713 ± 0.066 | 0.076 | 0.470 |
| IL-6/IL-13^3^ | 2.006 ± 0.052 | 1.979 ± 0.052 | 0.728 | 0.854 |
| IL-7/ IL-13^3^ | 0.276 ± 0.022 | 0.294 ± 0.017 | 0.510 | 0.833 |
| IL-12(p70)/IL-13^3^ | 1.384 ± 0.086 | 1.452 ± 0.063 | 0.523 | 0.833 |
| IL-17A/IL-13^3^ | 0.255 ± 0.019 | 0.246 ± 0.008 | 0.653 | 0.838 |
| IL18/IL-13^3^ | 5.338 ± 0.307 | 5.455 ± 0.372 | 0.819 | 0.911 |
| G-CSF/IL-13^3^ | 0.044 ± 0.003 | 0.043 ± 0.001 | 0.773 | 0.886 |
| GM-CSF/ IL-13^3^ | 0.282 ± 0.023 | 0.294 ± 0.013 | 0.632 | 0.836 |
| GRO/ KC / IL-13^3^ | 0.194 ± 0.024 | 0.198 ± 0.017 | 0.904 | 0.977 |
| IFNγ/IL-13^3^ | 1.699 ± 0.058 | 1.661 ± 0.037 | 0.569 | 0.836 |
| M-CSF/ IL-13^3^ | 0.17 ± 0.009 | 0.161 ± 0.008 | 0.447 | 0.817 |
| MIP-1α/IL-13^3^ | 0.082 ± 0.005 | 0.081 ± 0.003 | 0.916 | 0.977 |
| MIP-3Α/ IL-13^3^ | 0.07 ± 0.005 | 0.06 ± 0.002 | 0.065 | 0.455 |
| RANTES/ IL-13^3^ | 0.716 ± 0.089 | 0.819 ± 0.062 | 0.345 | 0.735 |
| TNFα/IL-13^3^ | 3.71 ± 0.2 | 3.231 ± 0.076 | **0.028** | 0.397 |
| VEGF/ IL-13^3^ | 0.094 ± 0.01 | 0.101 ± 0.006 | 0.561 | 0.836 |
| MCP-1/ IL-13^3^ | 1.092 ± 0.102 | 1.16 ± 0.091 | 0.624 | 0.836 |

^1^Data analyzed via unpaired Student’s t-test. Bold p values indicate p<0.05; italicized p values represent p<0.1.

^2^False discovery rate p value.

^3^One sample in the MeDi group was identified as an outlier using the ROUT method. Value = 332.32 for IL-13. Therefore, data from this animal could not be expressed as a ratio and included in analyses. MeDi group includes 10 animals except for IL-13 ratios. WD group includes 7 animals.

FDR, false discovery rate; SEM, standard error of mean; MeDi, Mediterranean diet (n=10/group); WD, Western Diet (n=7/group).
